# The Clinicogenomic Landscape of Induction Failure in Childhood and Young Adult T-Cell Acute Lymphoblastic Leukemia

**DOI:** 10.1200/JCO.22.02734

**Published:** 2023-04-25

**Authors:** David O'Connor, Jonas Demeulemeester, Lucia Conde, Amy Kirkwood, Kent Fung, Foteini Papaleonidopoulou, Gianna Bloye, Nadine Farah, Sunniyat Rahman, Jeremy Hancock, Caroline Bateman, Sarah Inglott, Jon Mee, Javier Herrero, Peter Van Loo, Anthony V. Moorman, Ajay Vora, Marc R. Mansour

**Affiliations:** ^1^ UCL Cancer Institute, University College London, London, United Kingdom; ^2^ Department of Haematology, Great Ormond Street Hospital for Children NHS Foundation Trust, London, United Kingdom; ^3^ The Francis Crick Institute, London, United Kingdom; ^4^ VIB-KU Leuven Center for Cancer Biology, Leuven, Belgium; ^5^ Department of Oncology, Laboratory for Integrative Cancer Genomics, KU Leuven, Leuven, Belgium; ^6^ CR UK & UCL Cancer Trials Centre, UCL Cancer Institute, UCL, London, United Kingdom; ^7^ Peter MacCallum Cancer Centre, Melbourne, VIC, Australia; ^8^ Sir Peter MacCallum Department of Oncology, The University of Melbourne, Melbourne, VIC, Australia; ^9^ South West Genomic Laboratory Hub, North Bristol NHS Trust, Bristol, United Kingdom; ^10^ The Children's Hospital at Westmead, Sydney, NSW, Australia; ^11^ Cancer Research UK Clinical Trials Unit, University of Birmingham, Birmingham, United Kingdom; ^12^ Department of Genetics, The University of Texas MD Anderson Cancer Center, Houston, TX; ^13^ Department of Genomic Medicine, The University of Texas MD Anderson Cancer Center, Houston, TX; ^14^ Wolfson Childhood Cancer Centre, Translational and Clinical Research Institute, Newcastle University, Newcastle upon Tyne, United Kingdom; ^15^ Department of Developmental Biology and Cancer, UCL Great Ormond Street Institute of Child Health, London, United Kingdom

## Abstract

Failure to respond to induction chemotherapy portends a poor outcome in childhood acute lymphoblastic leukemia (ALL) and is more frequent in T-cell ALL (T-ALL) than B-cell ALL. We aimed to address the limited understanding of clinical and genetic factors that influence outcome in a cohort of patients with T-ALL induction failure (IF).

**METHODS:**

We studied all cases of T-ALL IF on two consecutive multinational randomized trials, UKALL2003 and UKALL2011, to define risk factors, treatment, and outcomes. We performed multiomic profiling to characterize the genomic landscape.

**RESULTS:**

IF occurred in 10.3% of cases and was significantly associated with increasing age, occurring in 20% of patients age 16 years and older. Five-year overall survival (OS) rates were 52.1% in IF and 90.2% in responsive patients (*P* < .001). Despite increased use of nelarabine-based chemotherapy consolidated by hematopoietic stem-cell transplant in UKALL2011, there was no improvement in outcome. Persistent end-of-consolidation molecular residual disease resulted in a significantly worse outcome (5-year OS, 14.3% *v* 68.5%; HR, 4.10; 95% CI, 1.35 to 12.45; *P* = .0071). Genomic profiling revealed a heterogeneous picture with 25 different initiating lesions converging on 10 subtype-defining genes. There was a remarkable abundance of TAL1 noncoding lesions, associated with a dismal outcome (5-year OS, 12.5%). Combining TAL1 lesions with mutations in the MYC and RAS pathways produces a genetic stratifier that identifies patients highly likely to fail conventional therapy (5-year OS, 23.1% *v* 86.4%; HR, 6.84; 95% CI, 2.78 to 16.78; *P* < .0001) and who should therefore be considered for experimental agents.

**CONCLUSION:**

The outcome of IF in T-ALL remains poor with current therapy. The lack of a unifying genetic driver suggests alternative approaches, particularly using immunotherapy, are urgently needed.

## INTRODUCTION

T-cell acute lymphoblastic leukemia (T-ALL) is an aggressive malignancy comprising 10%-15% of childhood ALL.^1^ Although most children are cured, outcomes remain inferior to B-cell ALL (B-ALL), particularly in relapsed and refractory disease.^2^ Failure to respond to induction therapy, on the basis of morphologic assessment, has long been recognized as a predictor of poor outcome.^3^ Previously, we redefined induction failure (IF), demonstrating that a level of molecular minimal residual disease (MRD) above 5%, even in the absence of morphologic blasts, more accurately identifies IF, selecting 10% of T-ALL cases, 3-fold more than in B-ALL.^4^ Historically, IF in T-ALL was demonstrated to have a particularly dismal outcome with a 10-year survival rate of only 19% in a large international study.^3^ Although there has been an improvement in outcome with survival around 50% in contemporary trials,^4,5^ better treatments are clearly needed.

**CONTEXT**

**Key Objective**
What clinical and genomic factors predict occurrence and outcome of induction failure (IF) in childhood and young adult T-cell acute lymphoblastic leukemia (T-ALL)?
**Knowledge Generated**
Incidence of IF increases with age and is associated with an immature leukemia dominated by the early thymic precursor phenotype and HOXA genetic subtype. Outcome is worse in patients with leukemias of the TAL1 subtype or carrying mutations in the RAS or MYC pathways, indicating novel therapies are required in this group.
**Relevance *(S. Bhatia)***
The heterogeneity in the genetic landscape of T-ALL patients with IF and the very poor outcome with current therapy present a critical need for novel therapies in this population.*
  *Relevance section written by *JCO* Associate Editor Smita Bhatia, MD, MPH, FASCO.

Despite the preponderance of IF in T-ALL, little is known about the factors that predict resistance and influence outcome, largely because patients are taken off trial, limiting details of subsequent treatment and response. Although our group and others have identified genetic drivers of IF in B-ALL, facilitating use of targeted therapies,^4,6,7^ the genomic landscape of IF T-ALL remains undefined, restricting treatment to conventional agents. Furthermore, no genetic biomarker has been associated with poor outcome in T-ALL, limiting the potential for treatment stratification.

To address this, to our knowledge, we present the largest cohort of T-ALL IF reported, comprising all IF cases on two large multinational randomized trials, UKALL2003 and UKALL2011, which recruited 5,876 patients over a 15-year period. We used existing trial data, supplemented with additional clinical data, and combined whole-genome sequencing (WGS) and RNA-sequencing (RNAseq), to comprehensively characterize the clinical and functional genetic landscape of T-ALL IF.

## METHODS

### Patients

**FIG 1.**
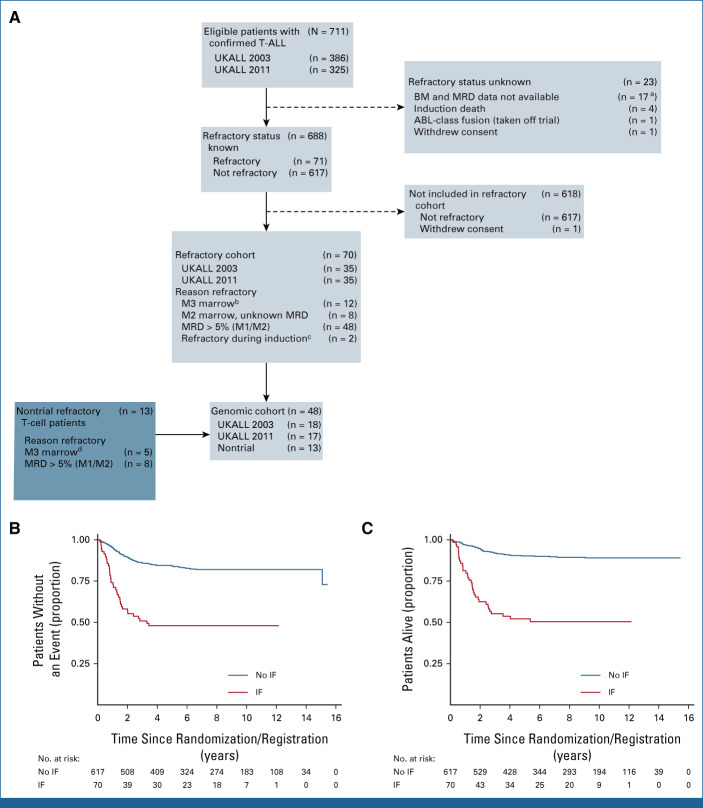
T-ALL IF cohorts and outcome. (A) Seventy-one patients of the 711 patients with T-ALL on the UKALL2003 and UKALL2011 trials suffered IF. One patient withdrew consent and was excluded from further analyses, leaving 70 cases in the trial cohort. Thirty-five of these patients were included in the genomics cohort with an additional 13 nontrial IF cases. (B) Kaplan-Meier curve showing event-free survival for patients with IF versus those who achieved remission on the UKALL2003 and UKALL2011 trials. (C) Kaplan-Meier curve showing overall survival for patients with IF versus those who achieved remission on the UKALL2003 and UKALL2011 trials. ^a^Includes 16 UKALL 2003 patients in CR but with no details and one UKALL 2011 patient with no data postregistration submitted. ^b^Any MRD. ^c^Rising WCC, switched treatment before D28. ^d^Any MRD. BM, bone marrow; CR, complete remission; IF, induction failure; MRD, minimal residual disease; T-ALL, T-cell acute lymphoblastic leukemia; WCC, white cell count.

The study uses two patient cohorts (Fig 1A). The trial cohort includes all 5,876 patients treated on the UKALL2003 and UKALL2011 trials, of whom 70 had T-ALL IF (combined median follow-up of 8.6 years [IQR, 5.5-10.5]). Both trials were conducted in accordance with the Declaration of Helsinki. Patients were enrolled at individual treatment centers by principal investigators after written informed consent from carers or patients was obtained. UKALL2003 was approved by the Scottish Multi-Centre Research Ethics Committee. UKAL2011 was approved by the North Thames Research Ethics Committee. The genomics cohort includes all patients with T-ALL IF from the trial cohort with samples available (n = 35) plus an additional 13 patients treated on nontrial protocols with identical induction therapy.

The responsive patient cohort used for comparison comprised 264 patients with T-ALL treated on the COG AALL0434 trial,^5^ all of whom responded to induction chemotherapy, that is, did not suffer IF. This group has been extensively characterized using a combination of WGS, whole-exome sequencing, RNAseq, conventional cytogenetics, and SNP array as previously reported.^8^ Samples were also subjected to targeted sequencing at noncoding hotspots.

IF was defined as end-of-induction (EOI) MRD ≥5%, irrespective of morphology, or an M2 or M3 marrow (morphologic blasts 5%-25% or >25%, respectively) without an MRD result, as per our previous study^4^ and reflecting the current definition used in contemporary clinical trials, such as the ALLTogether-01 study. Choice of subsequent therapy was discussed with the trial PI and left to the discretion of the treating center.

### UKALL2003 (ISRCTN Number 07355119) and UKALL2011 (ISRCTN Number 64515327)

UKALL2003 and UKALL2011 recruited children and young people (age 1-24 years) with ALL in the United Kingdom and Ireland between October 2003 and December 2018. The results of both UKALL2003^9,10^ and UKALL2011^11,12^ have been reported, with further details and full protocols available in the Data Supplement (online only).

Induction therapy was identical across both trials comprising dexamethasone, vincristine, pegylated L-asparaginase, and daunorubicin. In UKALL2003, patients with morphologic IF (M3 marrow) were taken off trial. From 2008, in recognition of poor prognosis, patients with MRD >10% were also recommended to be treated off trial. In UKALL2011, patients with M3 marrow and/or MRD high risk (day 29 >5% or week 14 >0.5%) were taken off trial.

### Samples

Samples were largely provided as frozen viable mononuclear cells, which were thawed and used for flow cytometric characterization to ascertain early thymic precursor (ETP) status, and DNA and RNA extraction.

### Sequencing

WGS was performed by Novogene Company Ltd (Cambridge) using Illumina sequencing to generate 150-base-pair paired-end reads on all samples at 50× coverage in tumor samples and 30× in germline samples. Total RNAseq was performed to generate 30 million 150-base-pair paired-end reads per sample.

Further details of sample processing, sequencing, and data analysis are provided in the Data Supplement.

## RESULTS

### Clinical Features

UKALL2003 and UKALL2011 recruited 5,876 patients between 2004 and 2019, of whom 711 (12.1%) had T-ALL; 688 were assessable at EOI. In total, 10.3% of patients with T-ALL experienced IF (n = 71; Fig 1A). Survival was significantly worse in IF (5-year event-free survival [EFS], 47.9% [95% CI, 35.8 to 59.1] *v* 84.0% [95% CI, 80.7 to 86.8]; HR, 4.14 [95% CI, 2.82 to 6.07]; *P* < .001; Fig 1B; 5-year overall survival [OS], 52.1% [95% CI, 39.7 to 63.1] *v* 90.2% [95% CI, 87.4 to 92.5]; HR, 6.38 [95% CI, 4.17 to 9.76]; *P* < .001; Fig 1C).

Multivariable assessment of factors associated with IF revealed a significant relationship with increasing age; IF occurred in 5.6% of patients younger than 10 years, 12.9% age 10-16 years, and 20% older than 16 years (*P* < .001; Data Supplement [Table A1]).

**FIG 2.**
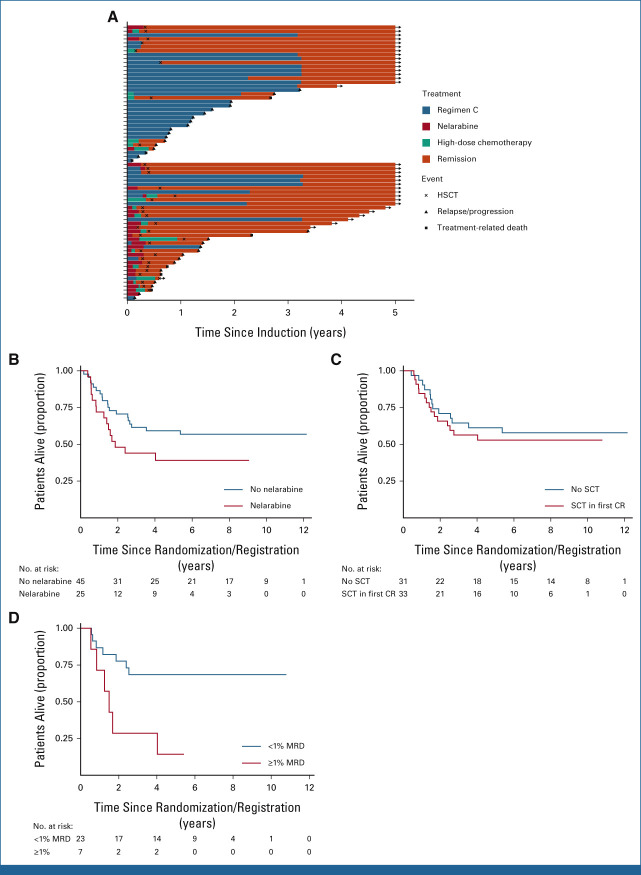
Treatment of T-ALL IF. (A) Swimmer plot displaying postinduction treatment of patients with IF split by trial. Standard chemotherapy indicates standard trial treatment. Nelarabine indicates nelarabine given either alone or in combination with other agents. High-dose chemotherapy indicates other non–nelarabine-containing regimens, most commonly FLA-IDA. (B) Kaplan-Meier plot showing OS of patients treated with nelarabine versus those not treated with nelarabine. (C) Kaplan-Meier plot showing OS of patients who received SCT versus those who did not. The SCT group included 33 patients (one excluded as underwent SCT when not in remission). The no SCT group included all 31 patients who remitted and were alive and in remission on day 91 (the date of the earliest SCT). (D) Kaplan-Meier plot showing OS of patients with end-of-consolidation MRD more than 1% versus MRD < 1%. CR, complete remission; FLA-IDA, fludarabine, cytarabine, idarubicin chemotherapy; HSCT, hematopoietic stem-cell transplantation; IF, induction failure; MRD, minimal residual disease; OS, overall survival; SCT, stem-cell transplant; T-ALL, T-cell acute lymphoblastic leukemia.

Since most patients with IF were treated off protocol, additional data were obtained on subsequent nontrial therapy from treating centers. Importantly, although patients failed to remit with induction therapy, only four patients never achieved remission, with all others responding to subsequent treatment. Postinduction therapy varied across the cohort but mainly followed two pathways, either continuation of the standard protocol or escalation to a nelarabine-containing regimen, most commonly in combination with cyclophosphamide and cytarabine as per COG AALL0434 consolidation,^5^ followed by hematopoietic stem-cell transplantation (HSCT; Fig 2A).

In line with international practice, there was a clear trend toward increasing use of nelarabine over time. Earlier patients treated on UKALL2003 commonly remained on standard trial chemotherapy (74.2%; 26/35) as most (63%; 22/35) were in a morphologic remission with molecular MRD > 5%; at this point, the IF definition did not include MRD and these patients were therefore considered to be in remission. By contrast, later patients, on UKALL2011, were frequently treated with nelarabine and HSCT (68.6%; 24/35; *P* = .0007). However, this did not translate into an improved outcome across the two trials (Data Supplement [Tables A2 and A3]). Accordingly, analyses of individual treatments showed no difference in outcome for patients who received nelarabine compared with those who did not (5-year OS, 39.1% [95% CI, 20.3 to 57.5] *v* 59.2% [95% CI, 43.3 to 72.0]; HR, 1.72 [95% CI, 0.88 to 3.42]; *P* = .11; Data Supplement [Table A4]) or those who underwent HSCT compared with those who did not (5-year OS, 52.8% [95% CI, 34.3 to 68.4] *v* 61.3% [95% CI, 42.0 to 75.9]; HR, 1.23 [95% CI, 0.58 to 2.58]; *P* = .59; Figs 2B and 2C). Although these were nonrandomized groups, with patients undergoing HSCT having higher levels of MRD (Data Supplement [Table A5]), the lack of improvement in outcome over time, despite the marked increased use of nelarabine and HSCT, suggests these approaches fail to overcome this high-risk disease. Notably, patients who relapsed after IF had a dismal outcome, with a median survival of only 3.9 months (IQR, 1.5-7.6; Data Supplement [Fig A1]).

Univariable and multivariable analyses of factors associated with outcome found no impact of age, white cell count, EOI morphology or MRD, or trial (Data Supplement [Tables A2 and A3]). However, a higher end-of-consolidation (EOC) MRD level was associated with inferior EFS (HR [1 log increase], 1.30 [95% CI, 1.06 to 1.55]; *P* = .011; Data Supplement [Table A6]). Patients with ≥1% EOC MRD had a 4-fold increased risk of death (5-year OS, 14.3% *v* 68.5%; HR, 4.10 [95% CI, 1.35 to 12.45]; *P* = .0071; Fig 2D) with only one of seven patients achieving long-term remission, despite all undergoing HSCT. By contrast, of the nine patients with MRD < 0.01%, eight of whom underwent HSCT, none relapsed but two died from HSCT-related mortality.

### Genetic Classification

Previously, we identified *PDGFRB* fusions as a major driver of B-ALL IF, allowing the use of targeted therapy.^4^ To identify analogous targetable lesions in T-ALL IF, we performed WGS on 48 cases (Fig 1A); 33 cases had paired germline material available, and 37 cases underwent RNAseq (Data Supplement [Table A7]). The outcomes and characteristics of the genomic cohort were representative of the full IF cohort (Data Supplement [Table A8 and Fig A2]).

**FIG 3.**
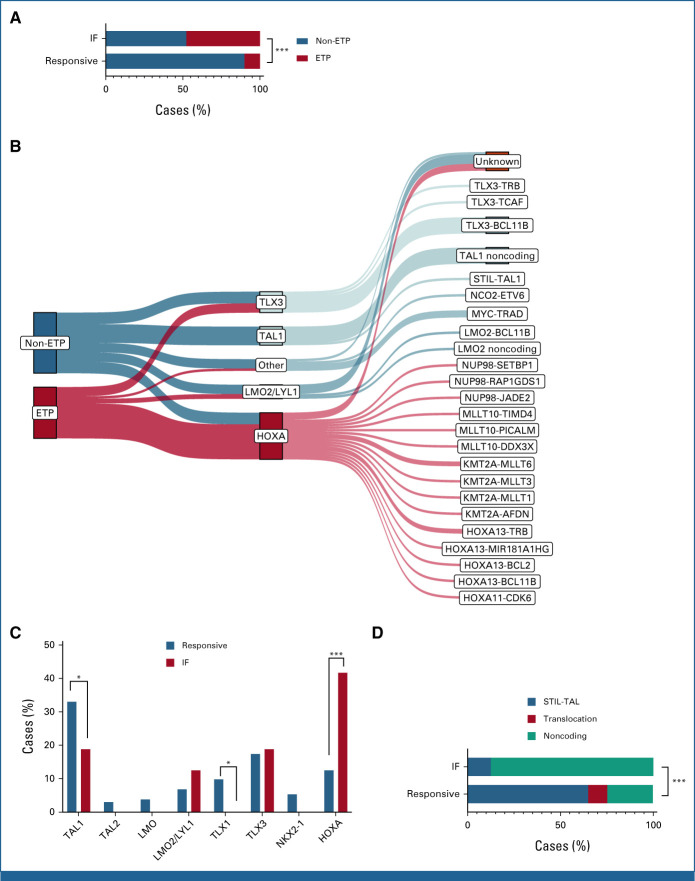
Phenotypic and genetic classification of T-ALL IF. (A) Proportion of flow cytometry–defined ETP phenotype in IF versus responsive cases. (B) Relationship between ETP status, genetic subgroup, and initiating genomic driver lesion. Note that all TAL1 cases co-occurred with either a LMO1 or LMO2 lesion, which is not shown in the figure. (C) Proportion of genetic subtypes in IF versus responsive cases of T-ALL. (D) Landscape of TAL1 driver lesion types in IF versus responsive cases of T-ALL. *P < .05, ***P < .001. ETP, early thymic progenitor; IF, induction failure; T-ALL, T-cell acute lymphoblastic leukemia.

We initially focused on classification of samples to conventional phenotypic and genetic subtypes. Flow cytometric analysis allowed identification of cases with an ETP phenotype, indicating a less differentiated, stem cell–like leukemia.^13^ Twenty-two of 46 cases (48%) with informative results had an ETP phenotype, significantly higher than the 10% of cases reported in responsive T-ALL (*P* < .001; Fig 3A).^8^

Historically, classification of T-ALL into genetic subtypes has relied on dysregulated expression of key transcription factor genes and hierarchical clustering of gene expression data.^14^ More recently, as with B-ALL and AML,^15,16^ there has been a progressive shift toward a system predicated on causative genomic lesions. WGS enables comprehensive interrogation of the genomic landscape permitting definitive allocation through detection of subtype-defining genetic lesions (Data Supplement [Fig A3]). Using this approach, initiating lesions were identified in 41 cases (85%), allowing allocation to conventional T-ALL genetic subgroups (Fig 3B). The genes involved, in decreasing order of frequency, were *TLX3* (n = 9), *TAL1* (n = 8), *LMO1* (n = 6), the *HOXA* locus (n = 6), *KMT2A* (n = 5), *LMO2* (n = 4), *MYC* (n = 4), *MLLT10* (n = 3), *NUP98* (n = 3), and *NUP214* (n = 3). A single case had an *ETV6*-*NCOA2* fusion, a previously described driver of T/myeloid leukemia.^17^ Notably, all *TAL1* cases co-occurred with either a *LMO1* or *LMO2* lesion, with *LMO1* lesions significantly more frequent in IF than in responsive cases (10.4% *v* 1.5%; *P* = .006). No driver lesion could be identified in the remaining seven cases, six of which could be allocated to genetic subgroups using RNAseq.

The relative proportion of genetic subtypes differed significantly between responsive and IF cases (Fig 3C). IF was restricted to the *HOXA*, *TAL1*, *TLX3,* and *LMO2*/*LYL1* subtypes, with almost half the cases allocated to the *HOXA* subtype, a significantly larger proportion than in responsive T-ALL (*P* < .001; Fig 3C). By contrast, there were significantly fewer *TAL1* cases (*P* = .026) with no *TLX1*, *TAL2,* or *NKX2-1* cases whatsoever, in keeping with the good prognosis reported in these subtypes. There was clear correlation between ETP status and genetic subtype; 75% of *HOXA* cases had an ETP phenotype, whereas 74% of *TAL1, LMO2/LYL1,* and T-other cases were non-ETP (*P* = .002).

Although the overall proportion of *TAL1* cases was lower in IF, there was an unexpected dominance of noncoding *TAL1* enhancer mutations. Although present in responsive cases, these only account for 24.6% of *TAL1* cases, with *TAL1* more commonly driven by the *STIL-TAL1* deletion.^8,18^ The converse is seen in the IF cases, with noncoding mutations accounting for 87.5% of *TAL1* cases, indicating a previously unrecognized link with treatment resistance (*P* < .001; Fig 3D). As characterized previously, the majority of mutations created novel binding sites for the transcription factor MYB (Data Supplement [Fig A4]).^18,19^

### Co-Operating Genetic Variants

**FIG 4.**
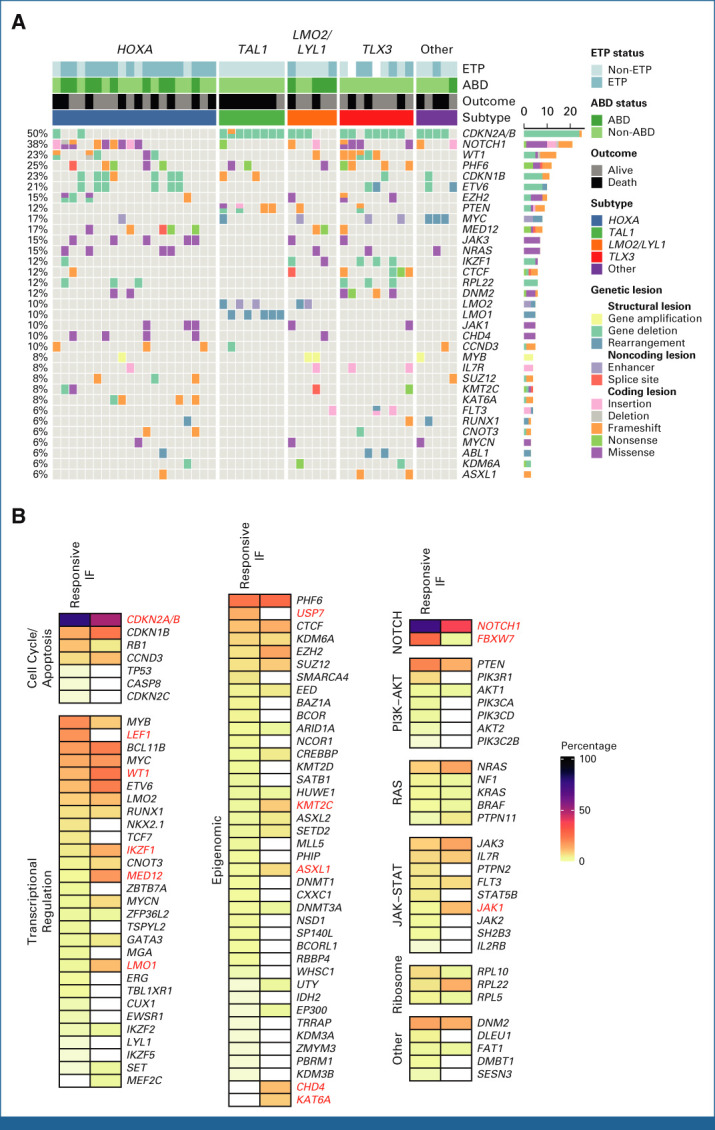
Mutational landscape of IF T-ALL. (A) Oncoplot showing somatic variants, ETP status, ABD status, outcome, and genetic subtype in IF T-ALL. (B) Frequency of mutations in IF versus responsive T-ALL. Names of genes showing a significant difference (*P* < .05) are in red. ABD, absence of biallelic deletion; ETP, early thymic progenitor; IF, induction failure; T-ALL, T-cell acute lymphoblastic leukemia.

There was a median of 21 coding, nonsynonymous SNV/indels (range, 4-144) per sample; the sample with the highest number of mutations had a somatic missense *MSH2* mutation, likely to result in acquired mismatch repair deficiency (Data Supplement [Tables A9-A11]). Driver gene discovery was limited to those previously reported in T-ALL or genes with mutations in more than two samples with germline available to confirm somatic status. As shown in Figure 4A, driver events occurred almost exclusively in known T-ALL genes, with the most frequent being *CDKN2A* (50%), *NOTCH1* (38%), and *PHF6* (25%). We found significantly higher mutation frequency in IF compared with responsive patients in two known T-ALL genes, *WT1* (22.9% *v* 11.4%; *P* = .037) and *MED12* (16.7% *v* 2.7%; *P* < .001; Fig 4B). By contrast, several highly recurrent T-ALL genes were less frequently mutated in IF, particularly *NOTCH1* (35.4% *v* 74.6%; *P* < .001), *FBXW7* (2.1% *v* 25.4%; *P* < .001), consistent with their previously reported association with good prognosis,^20,21^ and *CDKN2A* (50.0% *v* 78.4%; *P* < .001). *LEF1* and *USP7* are commonly mutated in T-ALL but we found no mutations in either gene in IF (0% *v* 17.4%; *P* < .001; 0% *v* 12.5%; *P* = .004, respectively; Fig 4B).

In addition, two genes not previously reported in T-ALL were recurrently mutated in IF. Five cases had mutations in the chromodomain-helicase-DNA-binding protein 4 (*CHD4*) gene, previously identified as a rare driver in B-ALL but not T-ALL,^22^ which encodes a core member of the nucleosome remodeling and deacetylase (NuRD) complex.^23^ Variants were clustered in a highly conserved region encompassing the helicase ATPase domain, in close proximity to variants reported as loss of function in endometrial cancer and Sifrim-Hitz-Weiss syndrome (Data Supplement [Fig A5]).^24,25^ Inspection of crystal structures of the nucleosome-CHD4 complex revealed mutations affect key amino acids involved in DNA binding and ATPase activity (Data Supplement [Fig A6]). Four of the five *CHD4* lesions occurred in *HOXA* cases, further supporting their functional relevance. A second gene, lysine acetyltransferase 6A (*KAT6A*), a histone acetyltransferase mutated in AML,^26^ harbored variants in four cases including a focal deletion and three truncating frameshift events likely to result in loss of function (Data Supplement [Fig A5]).

### Genomic Determinants of Outcome

**FIG 5.**
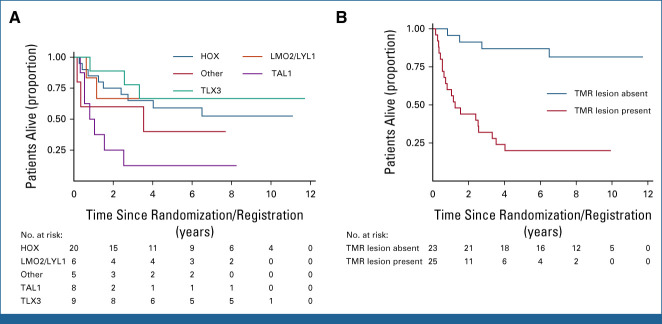
Genomic biomarkers of outcome in IF T-ALL. (A) Kaplan-Meier plot showing OS by genetic subtype. (B) Kaplan-Meier plot showing OS on the basis of the presence or absence of a mutation in the TAL1 gene or MYC or RAS pathways. IF, induction failure; OS, overall survival; T-ALL, T-cell acute lymphoblastic leukemia; TMR, TAL1/MYC/RAS.

Outcome differed significantly across genetic subtypes (Fig 5A; Data Supplement [Table A12]). *TAL1* cases had a particularly dismal outcome with OS of 12.5% (0.6-42.3). Notably, none of the seven patients with a *TAL1* noncoding driver lesion survived, and two never achieved remission, identifying this as a very high-risk genetic lesion in the context of IF. Despite the preponderance of ETP, the outcome was similar between ETP and non-ETP cases (Data Supplement [Fig A7]). Similarly, the absence of biallelic deletion of the *TRG* locus (ABD), an alternate means of identifying immature cases of T-ALL,^27^ showed no association with outcome (Data Supplement [Fig A7]).

Analysis of recurrently mutated genes showed significantly poorer survival in patients with mutations in *MYCN* and *NRAS* (Data Supplement [Fig A8]). Given the relatively small numbers of mutations in individual genes, we grouped genes by key oncogenic pathways (RAS, PI3K/AKT, IL7R/JAK, and MYC/MYCN), identifying significantly worse outcomes in patients with mutations in the RAS or MYC pathways (Data Supplement [Fig A9]). Since these mutations largely occur in the non-TAL1 subtypes (Fig 4A), selecting patients with a TAL1 lesion and/or mutations in the MYC or RAS pathways, which we term TMR lesions, allows division of the cohort into two groups with markedly different outcomes (Data Supplement [Table A13]). Those with a TMR lesion had a 5-year OS of only 23.1% compared with 86.4% in those without a TMR lesion (HR, 6.84 [95% CI, 2.78 to 16.78]; *P* < .0001; Fig 5B). Notably, six patients never achieved remission, all of whom were in the TMR group.

### Subclonal Landscape

The genomic results show a highly heterogeneous landscape of genetic variants, almost all of which are also seen in responsive disease. This raises the possibility that a refractory subclone, below the level of WGS detection, exists at presentation and expands through the selective pressure of induction therapy to become the dominant clone at EOI. To test this hypothesis, we performed WGS on three cases with EOI samples available. Although there was evidence of clonal heterogeneity at diagnosis, we did not observe clonal evolution over the course of induction, with only a single variant in *PTEN* lost in one case (Data Supplement [Fig A10]). Specifically, no new variants emerged, indicating that disease at presentation is representative of true refractory leukemia and that IF does not occur as a result of chemotherapy-induced mutagenesis.

## DISCUSSION

In this study, comprising over 700 children with T-ALL, we demonstrate that IF occurs in 10% of patients, with only half of this group achieving long-term survival, a dismal outcome in the context of pediatric ALL. IF was particularly common in older patients, with one in five older than16 years suffering IF, a previously unreported association highlighting the need to counsel this group at diagnosis of the potential risk of treatment failure.

Most disappointingly, we found no clear benefit of treatment intensification with nelarabine and HSCT, an approach that has been adopted as standard of care internationally.^28^ Although not a true randomization, the change in treatment strategy over the study period provides a temporal randomization, with no difference in outcomes across the two trials. This is in keeping with the outcomes of IF cases treated on the COG AALL0434 trial who were allocated nelarabine but achieved a 5-year EFS of only 53%, comparable with the outcome of our cohort.^5^ Although there was no clear benefit with HSCT, there was a higher disease burden at EOI in this group, making it possible that a subgroup of patients did derive benefit from HSCT. Addressing this in a randomized control trial is desirable but, in reality, unrealistic, given the number of patients required to power such a study. Although EOC MRD levels were only available in a subset of our cohort, this was a significant stratifier of outcome in IF, as has been shown in responsive T-ALL.^29^ Unsurprisingly, almost no patients with persistently high MRD after consolidation therapy survived. By contrast, in patients with very low MRD after consolidation, there were no relapses but two deaths due to HSCT, suggesting that patients may benefit from a chemotherapy-only protocol, removing the toxicity of HSCT.

In addition to EOC MRD, we identified several genetic biomarkers of poor outcome in the context of IF. Combining TAL1 lesions or mutations in the MYC and RAS pathways (TMR lesions) produces a gene set that identifies patients likely to fail conventional therapy and who should be considered for experimental agents. Although patients with RAS pathway lesions could be considered for targeted therapy, such as MEK inhibitors, the other lesions are not currently amenable to targeted therapy, and the sheer genetic diversity seen in T-ALL IF will make identification of effective agents challenging. Instead, our findings support the current focus on pathway-agnostic immunotherapy, such as chimeric antigen receptor T-cell (CAR-T) therapy targeting ubiquitous T-ALL antigens, such as CD7.^30^

The genomic analyses highlight the strength of WGS, painting a picture of marked genetic heterogeneity, with 25 different initiating lesions converging on 10 subtype-defining T-ALL genes, rather than a single unifying driver of refractory disease. The lack of a clear dominant driver is somewhat surprising. Our sequencing of samples at the EOI dismisses the possibility that a low-level treatment-resistant subclone exists at diagnosis and becomes dominant through the induction period, suggesting other nongenetic mechanisms may drive refractory disease, as described in AML.^31,32^

Biological classification shows a clear dominance of the ETP phenotype and HOXA genetic subtype, which are associated with a more stem-cell–/myeloid-like phenotype. This is consistent with the increased mutations in *WT1* (22.9% of cases), which is frequently mutated in AML and associated with poor prognosis.^33^ Notably, we did not find enrichment of genes and pathways previously implicated in high-risk disease such as *PTEN*, *RAS, PRC2,* and *TP53*. Interestingly, we found recurrent mutations in two novel T-ALL genes, *CHD4* and *KAT6A*, encompassing 20% of cases. *CHD4* mutations have previously been reported in AML and are particularly common in endometrial cancer.^34,35^ Inspection of crystal structures indicates variants are likely to interfere with DNA binding and ATPase function resulting in loss of function. *KAT6A* has essential roles in hematopoietic cells and is the target of recurrent translocations in AML.^36,37^ Characterization of the effect of these lesions on drug response in T-ALL is vital.

The landscape of *TAL1* lesions in IF is particularly striking. Although *TAL1* is the most common subtype of T-ALL, activation is predominantly through the *STIL*-*TAL* deletion with a minority caused by noncoding lesions.^8,18^ In the IF group, we observe a reversal of this ratio, with noncoding lesions the dominant driver of *TAL1* overexpression. Furthermore, these patients have a dismal outcome, with no long-term survivors and many failing to even achieve remission. To our knowledge, this is the first time a noncoding enhancer lesion has been found to affect prognosis, which should provide a stimulus for further study of the noncoding genome in other cancers. At present, we can only speculate on why alternate lesions that should simply result in *TAL1* overexpression can have such dramatically different effects on treatment response. For instance, a previous study found higher levels of *TAL1* expression in patients with noncoding lesions, which may reduce chemosensitivity in this group^38^; further work is required to explore this fascinating finding.

The abysmal outcome in those relapsing after IF emphasizes that there is only one opportunity to cure these patients and better therapies are urgently needed to achieve this. The lack of progress made in relapsed/refractory T-ALL over the past two decades contrasts starkly with the influx of efficacious immunotherapies in B-ALL.^39-41^ Thankfully, similar agents are now on the horizon for T-ALL with monoclonal antibodies and CAR-T cells entering the clinical arena.^30^

## Data Availability

The National Cancer Research Institute Children's Cancer and Leukemia Group Leukemia Subgroup will consider data sharing requests from researchers investigating questions regarding the biology and treatment of acute lymphoblastic leukemia. Data, including deidentified individual patient data, and study details will be released if the project is deemed pertinent. Initial requests should be directed to Dr David O'Connor (david.o'connor@ucl.ac.uk).
